# Venereal Prevention

**Published:** 1920-08-14

**Authors:** 


### THE EDITOR'S LETTER-BOX.

**Correspondence on all subjects is invited, but we cannot in any way be responsible for the opinions expressed by our correspondents, who must give their name and address as a guarantee of good faith, but not necessarily for publication. Correspondents are reminded that brevity of style and conciseness of statement greatly facilitate early insertion.**

### Venereal Prevention.

*To the Editor of* THE HOSPITAL.

SIR,—A correspondent whose letter is signed "Ali Baba" fiercely attacks (in your issue of July 31) certain of the propaganda of the Society for the Prevention of Venereal Disease on the ground that it is morally degrading. I hold no brief for that Society, of which I am not a member, but I ask leave to point out an inconsistency in "Ali Baba's" letter. He begins by proclaiming as his ideal the *total abolition* of venereal disease (italics his, not mine). He then details three "stepping-stones" as being "probably" necessary, and goes on to affirm that the raising of the standard of public morality is the thing most necessary to achieve the ideal. I do not quarrel with either his stepping-stones or his desire to raise the standard of morality, public or private. But I must object to his abuse of the Society for Prevention for advocating yet another—and, as they believe, very important—stepping-stone towards the achievement of total abolition. If the Society can show (I am not offering an opinion on the question) that "packet" prevention diminishes venereal diseases, clearly their leaflets are conducing to the abolition of those diseases. Nor is it fair to describe the packets as "inviting promiscuous sexual intercourse"; the innuendo is untrue, and is as morally degrading to "Ali Baba" as he thinks the pamphlets are to the Society.

As for the standard of morality, every decent citizen is in favour of the attempt to raise it. But there are two views of the prospect of a success sufficiently great to abolish venereal disease. Those who believe this possible are not, in my own view, facing facts. Because they may have been wholly continent themselves during celibacy, they must not assume that everybody else can be the same. In spite of much that has been written, and is no doubt honestly believed, by people who either have forgotten their youth or were always sexually subnormal, it does not alter the fact that for large numbers of the population complete sexual abstinence is impossible, in the literal sense of that word. To deny this or to shut one's eyes to it is to blink an important aspect of an intensely important question. Human nature being what it is, no amount of propaganda will abolish promiscuous intercourse; the teachings of Christianity, for example, have not made much impression in this respect during nineteen centuries. I submit, therefore, that to take this fact (as I believe it to be) into account when planning an anti-venereal campaign is good generalship, not an act of moral degradation; to refuse to recognise it seems to me fatal to that total abolition which cannot be any nearer the heart of "Ali Baba" than it is to that of yours faithfully,

August 6, 1920. "GENERAL PRACTITIONER."

